# Association Between COVID‐19 Vaccination and Gastrointestinal Manifestations: A Cross‐Sectional Study

**DOI:** 10.1002/hsr2.71231

**Published:** 2025-09-14

**Authors:** Mu'taz Massad, Mohanad Odeh, Shahed Al‐Ghsoon, Amr El‐Mousa, Haya Hindiyeh, Hala Hindiyeh, Ali Shahin, Jumana Sanwar, Hashem Abu Serhan, Abdulqadir J. Nashwan

**Affiliations:** ^1^ Department of Internal Medicine, Faculty of Medicine The Hashemite University Zarqa Jordan; ^2^ Department of Clinical Pharmacy and Pharmacy Practice, Faculty of Pharmaceutical Sciences The Hashemite University Zarqa Jordan; ^3^ Department of Ophthalmology Hamad Medical Corporation Doha Qatar; ^4^ Nursing & Midwifery Research Department (NMRD) Hamad Medical Corporation Doha Qatar

**Keywords:** adverse effects, COVID‐19, COVID‐19 vaccine, gastrointestinal

## Abstract

**Aim:**

To investigate a possible association between COVID‐19 vaccines and gastrointestinal manifestations.

**Methods:**

A cross‐sectional study employing convenience sampling was used to collect data using an online survey developed by the research team in Amman, Jordan. The study was web‐based and designed with Google Forms and was carried out between October 1, 2022, and February 1, 2023. Nine hundred eighty‐seven responses were included, aged 18 and above and had taken the COVID‐19 vaccine with their complete agreement to participate in the study, using the Chi‐square test and binary logistic regression models with (95%) confidence interval performed using SPSS version 25.0 (IBM Corp, Armonk, NY, USA). Statistical significance was set at *p* < 0.05.

**Results:**

Participants were 987 individuals; nearly half were between 18 and 25 years old, 65% were female, and 75% had bachelor's degrees. Seventy percent received two doses, with 37.5% reporting side effects. Females reported significantly more side effects than males (41.3% vs. 30.5%, *p* = 0.001); those with previous COVID‐19 infection reported higher side effects (41.6% vs. 34.7%, *p* = 0.028); and hospitalized individuals due to side effects had higher side effect rates (72.4% vs. 36.4%, *p* < 0.001). The second dose correlated with fewer side effects, with specific symptoms (indigestion, anorexia, flatulence, and abdominal pain) exhibiting significant differences (*p* < 0.05).

**Conclusion:**

This study found a positive association between COVID‐19 vaccines and gastrointestinal side effects, including significant heartburn, anorexia, and bloating.

## 1 Introduction

Severe acute respiratory distress syndrome coronavirus‐2 (SARS‐CoV‐2) is the cause of COVID‐19, the pandemic that conquered the world and caused over 3.4 million deaths worldwide [1]. Vaccines have always been used as a primary disease prevention measure and have been effective in curbing infection rates. Though SARS‐CoV‐2 vaccinations are usually well‐tolerated, side effects have been reported [2], these side effects ranged from mild to severe and rarely lethal [3], yet the use of the vaccine had benefits outweigh those adverse effects so far, there have been four vaccines being administered worldwide, developed by Pfizer‐BioNTech, Moderna, Sinopharm, Johnson & Johnson, where Pfizer‐BioNTech and Moderna worked on targeting the surface protein with mRNA vaccine and Johnson& Johnson used a technology triggering the immune response [4]. The potential side effects of the COVID‐19 vaccine are quite common, such as fatigue, headaches, and fever. Nevertheless, affected digestive symptoms—indigestion, bloating, and abdominal pain have been the reported ones, but they are not highly researched. However, despite the fact that many people were vaccinated worldwide, the discovery of any serious gastrointestinal side effects is of paramount importance for the vaccine's safety. This study is concerned with the relationship between COVID‐19 vaccination and gastrointestinal side effects in vaccinated individuals.

However, the prevalence of these vaccines' immediate and prolonged side effects is being studied; our study was done to demonstrate those side effects related to the body's gastrointestinal system promoted with the type of vaccine taken and the severity of each reported symptom in Jordan where the number of cases was 1,746,997 confirmed cases reported by WHO (World Health Organization) as of February 2023.

## 2 Materials and Methods

### 2.1 Study Design and Participants

This original research was performed according to the Declaration of Helsinki and COPE guidelines. Ethical approval was obtained from The Hashemite University (approval number: 6/3/2022/2023). Data was collected from an online anonymous survey from October 1, 2022, to February 1, 2023. Google Forms, an online survey platform, was used to publish and distribute the questionnaire randomly, allowing participants to fill it out only once voluntarily. Before completing the questionnaire, participants were informed that it was completely anonymous and voluntary, and that all data would be confidential. The URL for the survey was generated and then shared via social networks such as Facebook, Twitter, and WhatsApp, where people willing to fill it out can find the URL. Our study followed STROBE checklist recommendations (Supporting Information S1: File).

The inclusion criteria consist of (1) COVID‐19 vaccinated individuals, regardless of the number of doses administered, (2) voluntarily agreeing to participate in the online survey, and completing the self‐administered questionnaire independently. The exclusion criteria are (1) individuals aged less than 18 and (2) pregnancy.

Up until August 8, 2023, when this study started taking place, the Pfizer vaccine was safely administered to all children from the age of 5, and both Moderna and Pfizer vaccines are licensed for use in children from the age of 12 [5] (except those who are reported to have severe allergic reactions). The latest census has administered at least 12.5 billion doses of COVID‐19 vaccines so far, and 4.9 billion people have been fully vaccinated (two doses in the case of Pfizer, Moderna, AstraZeneca, Johnson, or Sinopharm) [6]. In Jordan, 10,007,983 doses of COVID‐19 vaccines have been administered as of February 2023.

### 2.2 Measures

We have implemented a comprehensive questionnaire to gather the essential data after conducting a literature search on different databases, including PubMed, ScienceDirect, and Google Scholar, making the questionnaire a critical component of our research. The questionnaire took into consideration only the gastrointestinal symptoms that people had to be more focused on them only. It is structured into three distinct parts, addressing various crucial aspects, including demographic information, vaccine details, health history, and reported post‐vaccine gastrointestinal side effects and symptoms; each side effect reported was evaluated based on a self‐reported scale of intensity and categorized into three classes: mild, moderate, and severe. All responses have been treated with the highest level of confidentiality and used exclusively for medical statistical purposes within the research.

### 2.3 Sample Size Calculation

The Jordanian population during the study's inception was approximately 11,302,000 as documented by the Jordan Department of Statistics for the year 2022 [7]. Thus, a sample size representative of the population, with a confidence level (CI) of 99% and a marginal error of 5%, was determined to be a minimum of 664 participants. This calculation aligns with the guidelines proposed by Taherdoost and was executed using a sample size calculator [8].

### 2.4 Statistical Analysis

For descriptive analysis, raw models will be unadjusted models. Adjusted models will include the age, sex, and type of vaccine to express the distribution of the manifestations and categorize them accordingly. Numbers, percentages, and tests will be used to detail sample characteristics and evaluate the associations between the number of doses received, the recipient's age, sex, and the possible gastrointestinal side effects experienced during and after the administration of the doses. Subsequently, this will allow us to study the bivariate association between the types of vaccine received and other independent variables, with the possible gastrointestinal side effects and their intensity (dependent variable). Since our hypothesis involves an association, a chi‐squared test (*X* ^2^) was used to assess the relationship. The Fisher's exact test was applied when the expected cell frequencies were less than 5.

To report the effect size along with the probability value, Phi (Phi φ) was used as a measure of association. Cramér's Phi (*φ*_c_) was applied when more than two variables were tested. The interpretation of Phi and Cramér's *V* was based on the threshold values recommended in Akoglu's 2018 study. Cramér's Phi is calculated by dividing the chi‐square value by the sample size and taking the square root. According to Akoglu, a Phi value of 1 indicates complete association, 0 indicates no association, values > 0.25 suggest a very strong relationship, values > 0.15 indicate a strong relationship, values > 0.1 suggest a moderate relationship, and values > 0.05 indicate a weak relationship.

All analyses were performed using SPSS version 25.0 (IBM Corp., Armonk, NY, USA), with statistical significance set at a two‐sided *p* value of < 0.05.

## 3 Results

Regarding the age of the participants, 52.9% were between 18 and 25, 14% were between 26 and 35, 10.8% were between 36 and 45, 13.7% were between 46 and 55, 7% were between 56 and 64, and 1.6% were above 65. 64.8% were female, the rest were male, 61.4% were single, 34.8% were married, and the rest were divided equally between divorced and widowed. 75.6% of the participants had completed their bachelor's degree. 59.6% of the participants had no previous known COVID‐19 infection, 55.3% had received the Pfizer vaccine, and 26.7% had the Sinopharm vaccine. Only 37.5% had general side effects after the vaccine, 2.9% of participants were hospitalized due to the side effects, and 79% of hospitalized patients lasted less than 1 week (Table 1).

**Table 1:** Participants' demographic characteristics.

Variable	Category	*N*	%
Age	18–25	522	52.9
	26–35	138	14.0
	36–45	107	10.8
	46–55	135	13.7
	56–64	69	7.0
	> 65	16	1.6
Gender	Female	640	64.8
	Male	347	35.2
Marital status	Widowed	19	1.9
	Single	606	61.4
	Married	343	34.8
	Divorced	19	1.9
Education	Secondary	104	10.5
	Bachelors	746	75.6
	Postgraduate level	137	13.9
History of COVID‐19 infection	No	588	59.6
	Yes	399	40.4
Number of vaccines	4	27	2.7
	3	231	23.4
	2	708	71.7
	1	21	2.1
Type of vaccine	AstraZeneca	66	6.7
	Johnson	1	0.1
	Sputnik	10	1.0
	Sinopharm	264	26.7
	Pfizer	546	55.3
	Received more than 1 vaccine	89	9.0
	Moderna	11	1.1
Side effects after vaccine	No	617	62.5
	Yes	370	37.5
Hospitalization due to side effects	No	958	97.1
	Yes	29	2.9
Hospitalization period	Less than 1 week	23	79%
	Less than 2 weeks	4	14%
	Less than 1 month	1	3%
	More than 1 month	1	3%

Statistically significant associations were observed with gender, where females reported a higher percentage of side effects (41.3%) than males (30.5%), *p* = 0.001. As for the history of COVID‐19 infection, 41.6% were infected with COVID‐19 before vaccination compared to the 34.7% that did not have a previous infection, *p* = 0.028. As expected, those who had been hospitalized due to side effects (72.4%) reported a higher percentage of adverse events than those who had not (36.4%), *p* < 0.001.

Pfizer was the most commonly administered vaccine (55.3% of participants), and it had a notable percentage of side effects reported (39.9% of those who received it). Sinopharm was the second most common vaccine (26.7%), with fewer participants reporting side effects (33%). Other vaccines like AstraZeneca (42.4%) and Moderna (45.5%) had higher percentages of side effects but were administered to fewer participants. Thus, while Pfizer caused the most side effects in absolute numbers due to the large number of people who received it, Moderna and AstraZeneca had higher relative percentages of participants reporting side effects (Table 2).

**Table 2:** Association and strength between demographic variables and the reported side effects.

Variable	Categories/side effects	No	Yes	Total	*X* ^2^	*p*	Phi, *φ*
Age	18–25	336	186	522	6.8	0.23	0.08
		64.4%	35.6%	100.0%			
	26–35	85	53	138			
		61.6%	38.4%	100.0%			
	36–45	59	48	107			
		55.1%	44.9%	100.0%			
	46–55	79	56	135			
		58.5%	41.5%	100.0%			
	56–64	45	24	69			
		65.2%	34.8%	100.0%			
	> 65	13	3	16			
		81.3%	18.8%	100.0%			
Gender	Female	376	264	640	11	0.001	0.11
		58.8%	41.3%	100.0%			
	Male	241	106	347			
		69.5%	30.5%	100.0%			
Marital status	Widowed	12	7	19	4.9	0.17	0.07
		63.2%	36.8%	100.0%			
	Single	395	211	606			
		65.2%	34.8%	100.0%			
	Married	199	144	343			
		58.0%	42.0%	100.0%			
	Divorced	11	8	19			
		57.9%	42.1%	100.0%			
Education	Secondary	75	29	104	5.4	0.06	0.07
		72.1%	27.9%	100.0%			
	Bachelors	453	293	746			
		60.7%	39.3%	100.0%			
	Postgraduate level	89	48	137			
		65.0%	35.0%	100.0%			
History of COVID‐19 infection	No	384	204	588	4.8	0.028	0.07
		65.3%	34.7%	100.0%			
	Yes	233	166	399			
		58.4%	41.6%	100.0%			
Number of vaccines	4	18	9	27	0.6	0.89	0.2
		66.7%	33.3%	100.0%			
	3	147	84	231			
		63.6%	36.4%	100.0%			
	2	440	268	708			
		62.1%	37.9%	100.0%			
	1	12	9	21			
		57.1%	42.9%	100.0%			
Type of vaccine	AstraZeneca	38	28	66	10.1	0.12	0.1
		57.6%	42.4%	100.0%			
	Johnson	0	1	1			
		0.0%	100.0%	100.0%			
	Sputnik	9	1	10			
		90.0%	10.0%	100.0%			
	Sinopharm	177	87	264			
		67.0%	33.0%	100.0%			
	Pfizer	328	218	546			
		60.1%	39.9%	100.0%			
	I received more than 1 vaccine	59	30	89			
		66.3%	33.7%	100.0%			
	Moderna	6	5	11			
		54.5%	45.5%	100.0%			
Hospitalization due to side effects	No	609	349	958	15.5	< 0.001	0.14
		63.6%	36.4%	100.0%			
	Yes	8	21	29			
		27.6%	72.4%	100.0%			

*Note: X* ^2^: chi‐squared test.

Table 3 presents descriptive frequencies of side effects following the first and second vaccine doses, categorized by severity as “no side effects,” “mild,” “moderate,” and “severe.” Participants reported the highest frequency of severe side effects for bloating (*n* = 76, 7.7%) and flatulence (*n* = 67, 6.8%) after the first dose. The most commonly reported mild side effect was anorexia after the first dose (*n* = 112, 12%). Heartburn emerged as the most frequently reported moderate side effect following the first dose, with a total of *n* = 132 (13.4%).

**Table 3:** Descriptive frequencies of side effects following the first and second vaccine doses.

Symptoms/severity (frequency (%))	No side effects	Mild	Moderate	Severe	Total
Indigestion (first dose)	789 (79.9%)	93 (9.4%)	87 (8.8%)	18 (1.8%)	987 (100%)
Indigestion (second dose)	827 (83.8%)	85 (8.6%)	48 (4.9%)	27 (2.7%)	987 (100%)
Heartburn (first dose)	737 (74.7%)	91 (9.2%)	132 (13.4%)	27 (2.7%)	987 (100%)
Heartburn (second dose)	756 (76.6%)	89 (9%)	105 (10.6%)	37 (3.7%)	987 (100%)
Dysphagia (first dose)	842 (85.3%)	75 (7.6%)	54 (5.5%)	16 (1.6%)	987 (100%)
Dysphagia (second dose)	861 (87.2%)	63 (6.4%)	41 (4.2%)	22 (2.2%)	987 (100%)
Anorexia (first dose)	740 (75%)	118 (12%)	85 (8.6%)	44 (4.5%)	987 (100%)
Anorexia (second dose)	787 (79.7%)	92 (9.3%)	75 (7.6%)	33 (3.3%)	987 (100%)
Gastrointestinal reflux (first dose)	768 (77.8%)	92 (9.3%)	81 (8.2%)	46 (4.7%)	987 (100%)
Gastrointestinal reflux (second dose)	787 (79.7%)	84 (8.5%)	75 (7.6%)	41 (4.2%)	987 (100%)
Bloating (first dose)	691 (70%)	107 (10.8%)	113 (11.4%)	76 (7.7%)	987 (100%)
Bloating (second dose)	723 (73.3%)	100 (10.1%)	110 (11.1%)	54 (5.5%)	987 (100%)
Flatulence (first dose)	688 (69.7%)	114 (11.6%)	118 (12%)	67 (6.8%)	987 (100%)
Flatulence (second dose)	728 (73.8%)	97 (9.8%)	109 (11%)	53 (5.4%)	987 (100%)
Constipation (first dose)	763 (77.3%)	93 (9.4%)	86 (8.7%)	45 (4.6%)	987 (100%)
Constipation (second dose)	795 (80.5%)	76 (7.7%)	76 (7.7%)	40 (4.1%)	987 (100%)
Diarrhea (first dose)	826 (83.7%)	81 (8.2%)	51 (5.2%)	29 (2.9%)	987 (100%)
Diarrhea (second dose)	834 (84.5%)	86 (8.7%)	46 (4.7%)	21 (2.1%)	987 (100%)
Abdominal pain (first dose)	738 (74.8%)	111 (11.2%)	95 (9.6%)	43 (4.4%)	987 (100%)
Abdominal pain (second dose)	781 (79.1%)	97 (9.8%)	70 (7.1%)	39 (4%)	987 (100%)
Jaundice (first dose)	889 (90.1)	50 (5.1%)	33 (3.3%)	15 (1.5%)	987 (100%)
Jaundice (second dose)	991 (92.3)	41 (4.2%)	20 (2%)	15 (1.5%)	987 (100%)

The bivariate analysis in Table 4, which examined the associations between side effects and symptoms in the first and second doses, revealed a notable trend indicating a higher percentage of participants reporting no side effects in the second dose. However, statistically significant differences were observed only for indigestion (*p* = 0.026), anorexia (*p* = 0.011), flatulence (*p* = 0.045), and abdominal pain (*p* = 0.020). Specifically, 79.9% of participants did not experience indigestion after the first dose, which increased to 83.8% after the second dose (*X* ^2^ = 4.9, *p* = 0.026). Similarly, 75% did not report anorexia following the first dose, with the percentage increasing to 79.7% after the second dose (*X* ^2^ = 6.4, *p* = 0.011). In the case of flatulence, 69.7% of participants were free from this symptom after the first dose, and the percentage increased to 73.8% after the second dose (*X* ^2^ = 3.9, *p* = 0.045). Finally, 74.8% did not report abdominal pain after the first dose, and the percentage rose to 79.1% after the second dose (*X* ^2^ = 5.3, *p* = 0.020). Mild side effects compared to moderate demonstrated a mixed trend; 8.6% of participants experienced mild indigestion after the second dose compared to 4.9% who experienced moderate indigestion (*X* ^2^ = 17.5, *p* ≤ 0.0001). As for heartburn, 9.2% of participants reported mild symptoms after the first dose compared to 13.4% who reported moderate symptoms (*X* ^2^ = 13.6, *p* = 0.0002). In the case of dysphagia, 7.6% of participants experienced mild symptoms after the first dose compared to 5.5% who experienced moderate symptoms (*X* ^2^ = 6.1, *p* = 0.01), while 6.4% of participants experienced mild symptoms after the second dose compared to 4.2% who experienced moderate symptoms (*X* ^2^ = 7.9, *p* = 0.005). Additionally, 12% of participants reported mild anorexia after the first dose compared to 8.6% of participants who reported moderate anorexia (*X* ^2^ = 9.1, *p* = 0.0026). In the case of diarrhea, mild symptoms were reported by 8.2% of participants after the first dose of the vaccine compared to 5.2% who reported moderate symptoms (*X* ^2^ = 11.5, *p* = 0.0007), while 8.7% reported mild symptoms after the second dose compared to 4.7% who reported moderate symptoms (*X* ^2^ = 21.2, *p* = 0.0001). Finally, 5.1% of participants experienced mild jaundice after the first dose compared to 3.3% who experienced moderate jaundice (*X* ^2^ = 6.1, *p* = 0.01), while 4.2% of participants experienced mild symptoms after the second dose compared to 2% who experienced moderate symptoms (*X* ^2^ = 11.9, *p* = 0.0005). Mild to severe side effects demonstrated a statistically significantly higher percentage across all side effect symptoms.

**Table 4:** Bivariate analysis to explore associations between side effects and symptoms in the first and second doses (associations between mild, moderate, and severe side effects).

Symptoms/severity (frequency (%))	No side effects, *n* (%)	*X* ^2^, side effects 1st to 2nd dose	*p*	Mild *n* (%)	Moderate *n* (%)	*X* ^2^, mild to moderate	*p*	Severe *n* (%)	*X* ^2^, mild to severe	*p*	Total
Indigestion (first dose)	789 (79.9)	4.9	0.026	93 (9.4)	87 (8.8)	0.3	0.5	18 (1.8)	70.1	< 0.0001	987 (100)
Indigestion (second dose)	827 (83.8)			85 (8.6)	48 (4.9)	17.5	< 0.0001	27 (2.7)	46.1	< 0.0001	987 (100)
Heartburn (first dose)	737 (74.7)	0.9	0.3	91 (9.2)	132 (13.4)	13.6	0.0002	27 (2.7)	45.3	< 0.0001	987 (100)
Heartburn (second dose)	756 (76.6)			89 (9)	105 (10.6)	2.3	0.13	37 (3.7)	29.4	< 0.0001	987 (100)
Dysphagia (first dose)	842 (85.3)	1.5	0.2	75 (7.6)	54 (5.5)	6.1	0.01	16 (1.6)	55.5	< 0.0001	987 (100)
Dysphagia (second dose)	861 (87.2)			63 (6.4)	41 (4.2)	7.9	0.005	22 (2.2)	29.7	< 0.0001	987 (100)
Anorexia (first dose)	740 (75)	6.4	0.011	118 (12)	85 (8.6)	9.1	0.0026	44 (4.5)	50.2	< 0.0001	987 (100)
Anorexia (second dose)	787 (79.7)			92 (9.3)	75 (7.6)	2.9	0.08	33 (3.3)	40.4	< 0.0001	987 (100)
Gastrointestinal reflux (first dose)	768 (77.8)	1.1	0.29	92 (9.3)	81 (8.2)	1.2	0.28	46 (4.7)	22.3	< 0.0001	987 (100)
Gastrointestinal reflux (second dose)	787 (79.7)			84 (8.5)	75 (7.6)	0.8	0.36	41 (4.2)	21.5	< 0.0001	987 (100)
Bloating (first dose)	691 (70)	2.5	0.11	107 (10.8)	113 (11.4)	0.2	0.6	76 (7.7)	7.6	0.006	987 (100)
Bloating (second dose)	723 (73.3)			100 (10.1)	110 (11.1)	0.79	0.37	54 (5.5)	19.4	< 0.0001	987 (100)
Flatulence (first dose)	688 (69.7)	3.9	0.045	114 (11.6)	118 (12)	0.11	0.7	67 (6.8)	17.4	< 0.0001	987 (100)
Flatulence (second dose)	728 (73.8)			97 (9.8)	109 (11)	1.2	0.28	53 (5.4)	18.1	< 0.0001	987 (100)
Constipation (first dose)	763 (77.3)	3.1	0.07	93 (9.4)	86 (8.7)	0.25	0.6	45 (4.6)	24.1	< 0.0001	987 (100)
Constipation (second dose)	795 (80.5)			76 (7.7)	76 (7.7)	0	1	40 (4.1)	15.9	0.0001	987 (100)
Diarrhea (first dose)	826 (83.7)	0.2	0.6	81 (8.2)	51 (5.2)	11.5	0.0007	29 (2.9)	37.2	< 0.0001	987 (100)
Diarrhea (second dose)	834 (84.5)			86 (8.7)	46 (4.7)	21.2	0.0001	21 (2.1)	60.6	< 0.0001	987 (100)
Abdominal pain (first dose)	738 (74.8)	5.3	0.02	111 (11.2)	95 (9.6)	2.1	0.14	43 (4.4)	43.4	< 0.0001	987 (100)
Abdominal pain (second dose)	781 (79.1)			97 (9.8)	70 (7.1)	7.3	0.06	39 (4)	36.4	< 0.0001	987 (100)
Jaundice (first dose)	889 (90.1)	3.04	0.08	50 (5.1)	33 (3.3)	6.1	0.01	15 (1.5)	28.1	< 0.0001	987 (100)
Jaundice (second dose)	911 (92.3)			41 (4.2)	20 (2)	11.9	0.0005	15 (1.5)	18.9	< 0.0001	987 (100)

## 4 Discussion

Our study revealed that gastrointestinal side effects following COVID‐19 vaccination were relatively common, with heartburn, bloating, and anorexia being the most frequently reported. Other symptoms, including abdominal pain, diarrhea, nausea, and vomiting, were also observed, though at lower rates. These findings add to the limited literature on vaccine‐related digestive effects, which are often underreported or grouped under systemic reactions. Moreover, an analysis of intensity and prevalence revealed an intriguing pattern, exhibiting higher prevalence and intensity after the initial vaccine dose than subsequent doses [9], which suggests a potential acclimatization effect, where the body becomes less responsive to vaccine‐related side effects after the first exposure. Meanwhile, a diagnosis of vaccine‐related multiple organ inflammation, including liver, was reported after a booster dose in a previously healthy female who only had fever after the first two doses [10].

Among participants with prior COVID‐19 infection, gastrointestinal symptoms were more prevalent, supporting the hypothesis of an immune priming effect or “vaccine‐induced COVID‐19 mimicry,” where the vaccine elicits a response similar to that observed during infection [11, 12, 13]. However, this association did not extend to symptom severity, suggesting that prior infection influences susceptibility, but not necessarily outcome intensity [14].

Gender differences were also evident, with females reporting significantly more side effects than males (41.3% vs. 30.5%). This trend has been echoed in studies evaluating both primary and booster doses [15, 16], and may relate to hormonal, immunologic, or psychosocial factors. The role of the nocebo effect should also be considered, as clinical trials have demonstrated that negative expectations can amplify perceived side effects, with nocebo responses observed in up to 16.4% of participants [17, 18].

While the overall incidence of severe complications was low, our study identified that 2.9% of participants required hospitalization due to reported side effects. Additionally, some case studies reported rare yet significant complications, including acute diverticulitis [19], IgA vasculitis [20], acute pancreatitis [21], and acute acalculous cholecystitis [22]. These reported cases indicate the need for vigilant postvaccination monitoring in some patients [23].

## 5 Limitations

Defining the relationship between gastrointestinal issues and COVID‐19 vaccination poses several challenges. First, the symptoms reported could stem from factors beyond vaccination, complicating the analysis of causation. Additionally, as this is an observational study, biases are present, as participants self‐report their symptoms. This self‐reporting may lead to overestimations or underestimations of the severity of symptoms, potentially confusing post‐vaccine manifestations with pre‐existing conditions. Recall bias is another concern, as participants may inadvertently omit crucial details about their symptoms due to fear or embarrassment, which can significantly affect the accuracy of the information collected. Furthermore, a notable limitation of this study is the lack of clarity regarding the onset of symptoms. Without precise data on when symptoms began after vaccination, it becomes challenging to establish a timeline of events that accurately reflects the relationship between the vaccine and the reported gastrointestinal issues. This gap in information can hinder our understanding of the immediate and delayed effects of the vaccine on gastrointestinal health.

## 6 Conclusions

This study found a possible positive association between COVID‐19 vaccines and gastrointestinal side effects, including heartburn, anorexia, and bloating. Further research with larger samples is needed to confirm these findings and improve vaccine safety.

## Author Contributions

**Mu'taz Massad:** conceptualization, methodology, writing – review and editing, writing – original draft, data curation, formal analysis. **Mohanad Odeh:** methodology, data curation, writing – original draft, writing – review and editing. **Shahed Al‐Ghsoon:** methodology, data curation, writing – original draft, writing – review and editing. **Amr El‐Mousa:** methodology, data curation, writing – original draft, writing – review and editing. **Haya Hindiyeh:** data curation, methodology, writing – original draft, writing – review and editing. **Hala Hindiyeh:** methodology, data curation, writing – original draft, writing – review and editing. **Ali Shahin:** methodology, data curation, writing – original draft, writing – review and editing. **Jumana Sanwar:** methodology, data curation, writing – original draft, writing – review and editing. **Hashem Abu Serhan:** methodology, writing – review and editing, supervision. **Abdulqadir J. Nashwan:** methodology, writing – review and editing, supervision.

## Ethics Statement

This original research was performed according to the Declaration of Helsinki and COPE guidelines. Ethical approval was obtained from The Hashemite University (approval number: 6/3/2022/2023).

## Consent

Before completing the questionnaire, participants were informed that it was completely anonymous and voluntary, and that all data would be confidential.

## Conflicts of Interest

Abdulqadir J. Nashwan is an Editorial Board member of Health Science Reports and a coauthor of this article. To minimize bias, they were excluded from all editorial decision‐making related to the acceptance of this article for publication. The other authors declare no conflicts of interest.

## Transparency Statement

The lead author Abdulqadir J. Nashwan affirms that this manuscript is an honest, accurate, and transparent account of the study being reported; that no important aspects of the study have been omitted; and that any discrepancies from the study as planned (and, if relevant, registered) have been explained.

## Supporting information

STROBE‐checklist‐v4‐cross‐sectional Dr Motazz Side effects Vaccines.

## Data Availability

The data sets used and analyzed during the current study are available from the corresponding author upon reasonable request.
